# The effect of the China-Thailand railway on Thailand’s trade with China Guangxi Province

**DOI:** 10.1371/journal.pone.0350972

**Published:** 2026-09-18

**Authors:** Siyuan Wei, Liu Xia

**Affiliations:** 1 School of Logistics Management and Engineering, Nanning Normal University, Nanning City, Guangxi Province, China; 2 School of Culture and Tourism Management, Guangxi Technological College of Mechanical and Electrical, Nanning City, Guangxi Province, China; University of Sargodha, PAKISTAN

## Abstract

This study assesses how the China-Thailand Railway may reshape Thailand’s trade with Guangxi by altering the relative competitiveness of road, rail, and sea freight. A mixed-methods framework is employed, combining literature and secondary data with expert interviews, the Analytic Hierarchy Process (AHP), a multinomial Logit model of freight mode choice, and a SWOT analysis. Eight experts from customs, academia, logistics enterprises, trading companies, and railway construction evaluate six route- choice criteria: transportation time, transportation cost, convenience, safety, punctuality, and transportation capacity. AHP results show that cost and time jointly account for about 62% of decision weight, with capacity and punctuality forming a second tier. Using these weights and calibrated attributes, the Logit model estimates that, under a plausible operating scenario, sea, rail, and road would capture roughly 37.0%, 33.2% and 29.8% of freight, respectively. The findings indicate that the China-Thailand Railway can become a strong competitor to existing modes and, under appropriate policy and operational conditions, support more efficient, reliable, and sustainable Thailand-Guangxi trade.

## 1. Introduction

With the establishment of the ASEAN Free Trade Area and the signing of the Regional Comprehensive Economic Partnership (RCEP), China has deepened economic ties with Southeast Asian economies, creating new momentum for trade and investment integration. Within this broader regional context, China and Thailand have emerged as key trading partners, with bilateral trade reaching 887.6 billion yuan in 2023, while Thai-Guangxi trade accounted for 40.4 billion yuan, only 4.55% of the total (General Administration of Customs of the People’s Republic of China (GAC). Despite Guangxi’s geographic proximity and its formal role as a strategic bridgehead for China-ASEAN connectivity, most Thailand-China trade still relies on maritime routes via coastal provinces such as Guangdong, including ports in Guangzhou, Foshan, and Zhanjiang. Overland routes like R9 through Laos and Vietnam remain under-utilized due to poor road conditions, higher logistics costs, and operational uncertainty, underscoring the need for more efficient, reliable inland corridors.

Recent corridor initiatives, most notably the International Land-Sea Trade Corridor (ILSTC) centered on the Beibu Gulf ports and the China-Singapore International Land-Sea Trade Corridor, have shown that well-designed multi modal networks can significantly reshape freight flows between China and ASEAN countries by reducing transit times, logistics costs, and uncertainty [1]. Empirical studies on ILSTC highlight its potential to alter freight market structures across Western China, Central Asia, ASEAN, and even the EU by shifting market share toward newly created rail-sea options [2]. For Guangxi specifically, the ILSTC and New Western Land-Sea Corridor have been identified as critical levers for upgrading its logistics functions and deepening hinterland connectivity. Yet, the province’s direct trade with Thailand remains modest relative to its strategic position [3]. Parallel developments such as the China-Laos Railway have already begun to transform regional transport patterns, demonstrating measurable improvements in transit time and logistics performance for Thailand- China trade routed via Laos [4]. Building on these experiences, the planned China- Thailand Railway, linking Bangkok to Kunming and, via domestic rail, to Nanning, has the potential to create a new, time-competitive “golden corridor” between Thailand and Guangxi, with implications for both modal choice and trade structure [5,6].

However, the academic literature to date has focused primarily on the macro-level trade and network effects of new corridors such as ILSTC or the China-Laos Railway, often at the national or multi-country scale. Existing work has examined, for example, how ILSTC and related land-sea initiatives reconFig freight structures, trade patterns, and regional economic linkages across China, ASEAN, and Europe, using simulation or multinational Logit frameworks at the corridor level [7]. Other studies analyze China-Laos Railway-induced changes in Thailand’s export potential to China using linear regression and scenario-based projections [8]. Yet there is still very limited research that zooms in on the specific trade relationship between Thailand and Guangxi, and almost no studies that explicitly evaluate how the completion of the China-Thailand Railway would affect Thailand-Guangxi trade volumes and modal shares relative to existing sea and road options. This leaves an important empirical and policy gap: while Guangxi is formally positioned as a gateway to ASEAN under the Belt and Road and ILSTC frameworks, its role in Thailand-China trade, and the way a new rail corridor might reshape that role, remains under-examined.

Methodologically, transport and logistics research increasingly relies on multi-criteria decision-making (MCDM) tools to capture complex trade-offs among cost, time, reliability, safety, and capacity in infrastructure and mode-choice evaluation. Comprehensive reviews show that the Analytic Hierarchy Process (AHP) has become one of the most widely used MCDM methods in the transport sector, especially when stakeholder or expert judgements are essential [9,10]. Recent work documents how AHP and fuzzy AHP are applied to freight mode and route selection, terminal planning, and strategic logistics decisions in different regional contexts [11]. Earlier studies also demonstrate that AHP can be combined with discrete choice models to structure mode- choice problems and derive attribute weights that feed into utility-based formulations [12]. In parallel, multinomial logit and related discrete choice models remain the standard analytical tools for quantifying how shippers and logistics providers allocate flows across competing freight modes and corridors based on generalized costs and service attributes.

Against this backdrop, this paper focuses specifically on the prospective impact of the China-Thailand Railway on Thailand’s trade with Guangxi. It does so by combining expert-based AHP with a scenario- driven Logit framework, using updated expert judgements on six key criteria, transport cost, time, convenience, safety, punctuality, and capacity, to derive consistent criteria weights, and then embedding these weights into a multinomial Logit model that estimates route choice probabilities for alternative sea, road, and rail options. By integrating corridor-level insights from the ILSTC and China-Laos experiences with a focused, Guangxi-centered analysis of Thailand-China trade, the study contributes to three strands of literature: (i) empirical evaluation of new rail corridors’ effects on regional trade and freight structures, (ii) applications of AHP and MCDM in transport and logistics planning, and (iii) the use of AHP-informed Logit models to analyses freight mode choice under competing multimodal corridor scenarios. The findings are expected to inform both Chinese and Thai policymakers and logistics stakeholders on how to leverage the China-Thailand Railway to strengthen Thailand-Guangxi trade while optimizing mode allocation across sea, road, and rail.(1) This study identifies the core criteria influencing the selection of transportation modes for cross-border trade between Thailand and Guangxi, and determines their relative weightings through systematic evaluation. (2) It examines how the construction and operational launch of the China–Thailand Railway will reshape the market share distribution and competitive dynamics among road, maritime, and rail freight transport sectors in Thailand–Guangxi trade logistics. (3) It conducts a comprehensive SWOT analysis—assessing strengths, weaknesses, external opportunities, and strategic challenges—of the China–Thailand Railway’s role in optimizing Thailand–Guangxi trade logistics, and formulates targeted, actionable policy and operational recommendations.

## 2. Literature review

### 2.1. China-Thailand railway

The China-Thailand Railway is a flagship project under the Belt and Road Initiative, designed to serve as a core land corridor linking China with mainland Southeast Asia. Strategically, it is expected to strengthen China-ASEAN connectivity, reduce transport costs and transit times, and support deeper economic, social, and cultural exchanges among participating economies. The project also reflects a broader shift from maritime-dominated routes to more balanced multimodal and land-based corridors in China-ASEAN trade

The first phase of the Thai-Chinese high-speed railway, connecting Bangkok and Nakhon Ratchasima, is approximately 253 km long and designed for a speed of 250 km/h. Construction started after the groundbreaking ceremony held in Nakhon Ratchasima on December 21, 2017, with China providing design and technical support and Thailand responsible for financing and implementation under a public- private partnership framework. This line represents Thailand’s first standard-gauge high-speed railway and is conceived as the southern segment of a longer corridor linking Bangkok with Kunming through Laos.

On February 4, 2025, the Thai cabinet approved the second phase of the high-speed rail project from Nakhon Ratchasima to Nong Khai. The second phase is planned to be about 357 km long and includes the construction of a freight transfer center in Nong Khai to connect Thailand’s meter-gauge railway with the standard-gauge China-Laos Railway. The transfer center is intended to provide a “one-stop” service for international rail freight and facilitate seamless transshipment between different gauges. According to the approved plan, the second phase is scheduled to be completed around 2030–2031, at which point the Thai section will form a continuous high-speed rail corridor from Bangkok to the Lao border and, via the China-Laos Railway, to Kunming and further north.

Once fully operational, the China-Thailand Railway is expected to offer shorter end-to-end transit times than sea transport, lower costs than long-distance road transport, and significantly higher transport capacity. These features suggest that the railway could alter the relative competitiveness of existing modes, induce a partial shift from road and sea to rail, and reshape trade and logistics patterns between Thailand and Guangxi, where Nanning and the Beibu Gulf ports serve as key nodes in China-ASEAN connectivity. Existing studies on related corridors, such as the International Land-Sea Trade Corridor and the China- Laos Railway, already indicate that new rail and rail-sea routes can significantly reduce transit times and logistics costs and change freight allocation patterns across modes and corridors. Building on these insights, the present study focuses specifically on the Thailand-Guangxi relationship in the context of the China-Thailand Railway.

### 2.2. Influencing factors of overseas infrastructure

To identify the main factors through which large-scale overseas infrastructure projects affect national and regional development, this study reviews recent literature on international railways, ports, and logistics corridors. The focus is on empirical and conceptual studies published in the last five to seven years that examine trade, logistics, and the broader development impacts of infrastructure built under initiatives such as the Belt and Road, the International Land-Sea Trade Corridor, and related cross-border projects. Based on this review, six broad categories of influencing factors are identified: economy and trade, logistics, tourism, environment, security, and other factors (including human resources and institutional conditions). Table 1 summarizes the factors considered in 30 selected studies that analyze overseas infrastructure projects and their impacts. The sample includes work on overseas rail corridors, port development, cross- border economic zones, and regional connectivity projects in different countries and regions. For each study, the table records whether the analysis explicitly considers economy and trade, logistics, tourism, environment, security, or other relevant factors such as human resources, institutional quality or regional development.

**Table 1:** Main influencing factors selected in studies on overseas infrastructure.

Study (author & year)	Economy & trade	Logistics	Tourism	Environment	Security	Other
Su Qingxu [13]	✓	✓		✓		
Luo Mingran [14]	✓		✓			✓
Hao et al. [15]	✓		✓	✓		
Warinphat Accadachanon [16]	✓	✓		✓		
Liu and Jiang [17]	✓			✓		
Qin and Li [18]	✓					✓
Yi et al. [19]	✓	✓				✓
Huang et al. [20]		✓		✓		
Shao et al. [21]				✓		
Duan et al. [22]		✓				
MAO [23]		✓				
Ye and Cai [24]	✓					
Ma [25]	✓					✓
Yongjun et al. [26]	✓	✓		✓		
Lauridsen [27]	✓	✓		✓		
Liao and Katada [28]	✓	✓		✓		
Qin Lei [18]		✓				
Jiahui and Jinfei [29]	✓	✓		✓		
Wu & Chen [30]	✓	✓			✓	
Chun-meng [31]	✓	✓			✓	
Xu et al. [32]	✓		✓			
Li Lincan [33]	✓	✓				
Li Ruixue [34]	✓	✓				
Xie et al. [35]	✓	✓				
Qing and Tao [36]	✓					
Chen et al. [37]	✓		✓			
Liu Zecong [38]	✓	✓			✓	
Guo et al. [39]		✓			✓	
Jia Shuangying [40]		✓			✓	
Wang Xiaonan [41]		✓				
Bharti and Kumari [42]		✓			✓	
**Total (number of studies)**	**21**	**21**	**4**	**9**	**7**	**4**

Table 1 is independently compiled by the authors through systematic coding of the 30 peer-reviewed studies listed in the reference section; each literature’s research focus is manually recorded and counted.

Note: “Other “ primarily covers human resources, institutional, and regional development factors.

Among the 30 studies, economy, trade, and logistics each appear in 21 cases, indicating that these two dimensions are most commonly emphasized in evaluations of overseas infrastructure. Environmental impacts are discussed in 9 studies, while security-related issues and other factors (such as human resources and institutional capacity) appear in 7 and 4 studies, respectively. Tourism is explicitly considered in 4 studies, typically in the context of high-speed rail and cross-border tourism flows.

Overall, the literature suggests that overseas infrastructure projects affect multiple dimensions of national and regional development, but that economic performance, trade, and logistics are most frequently highlighted. In the context of the China-Thailand Railway and its implications for Guangxi, these findings support the decision to focus on the economy, trade, and logistics as primary dimensions in the broader conceptual framework, and to treat tourism, the environment, security, and other factors as secondary yet relevant considerations. The expert interview design and the subsequent SWOT analysis in this study build on this literature by structuring expert judgements around these six broad categories [43,44].

### 2.3. Bilateral trade between Thailand and Guangxi

Guangxi plays a dual role in China’s opening-up strategy: it is both a key gateway for the southwest region and a frontline platform for China-ASEAN cooperation. In 2024, Guangxi’s total imports and exports reached 756.39 billion yuan, a year-on-year increase of 9.4%, while trade with ASEAN amounted to 397.82 billion yuan, up 17.2%. ASEAN has remained Guangxi’s largest trading partner for many years, reflecting the growing intensity of regional integration. Nevertheless, when compared with China’s overall trade with ASEAN, Guangxi’s share remains modest, indicating considerable room for further expansion. The structure of trade between Thailand and Guangxi reflects the comparative advantages of both sides. Thailand’s exports to Guangxi are dominated by: (1) computers and related components, including automatic data processing equipment and integrated circuit boards; (2) agricultural products such as fresh and frozen shrimp, durian and mangosteen; (3) textiles and jewelry, including garments, jewelry, gemstones and primary chemical fibers; (4) rice and rubber, including high-grade rice and natural rubber; and (5) machinery, equipment and automotive parts. Guangxi’s exports to Thailand, in turn, are concentrated in: (1) agricultural products such as palm oil, bananas and fruit and vegetable juices; (2) machinery, equipment and automotive parts; (3) electronic products, including industrial electronics and integrated circuits; (4) chemical products, including various chemical materials and fuels; and (5) sugar and related products from the sugar industry. This pattern underscores the complementarities between Thailand’s agro-industrial base and Guangxi’s manufacturing and processing strengths.

Table 2 summaries the scale of Guangxi’s trade with ASEAN, particularly with Thailand and Vietnam. Although the exact values depend on the statistical source and currency conversion, the table clearly shows that Guangxi’s trade with ASEAN accounts for only a small share of China’s total trade with the region, and that Guangxi-Thailand trade within Thailand-China trade is relatively low. By contrast, Guangxi’s trade with Vietnam accounts for a much higher share of China-Vietnam trade.

**Table 2:** Import and export trade volume between Guangxi and ASEAN in 2024 (Unit: billion US dollars).

Partner	Guangxi exports	Guangxi imports	Guangxi’s total trade	China’s total trade with partners	Guangxi’s share in China’s trade (%)
ASEAN	288.64	106.18	558.74	9,823.40	5.7
Thailand	26.51	25.29	51.80	1,339.82	3.9

Data source: General Administration of Customs of China (2024) official cross-border trade statistical yearbook; currency conversion adopts the 2024 annual average USD-RMB central parity rate issued by the People’s Bank of China; minor rounding discrepancies exist in total Figs due to unit conversion.

Note: Figs are compiled from official customs statistics; minor discrepancies in totals may arise from rounding and currency conversion.

From the perspective of this study, two points are critical. First, Guangxi’s trade with ASEAN is growing rapidly but still accounts for only a relatively small share of China’s total trade with the region. Second, within this context, Guangxi’s bilateral trade volume with Thailand remains limited compared with its trade with Vietnam and other ASEAN members. This implies substantial growth potential if logistics constraints can be alleviated and new corridors, such as the China-Thailand Railway, can effectively reduce transport costs and times and improve reliability.

The Thailand-Guangxi trade structure, combined with Guangxi’s role as a gateway region and the current dominance of sea transport, underscores the need to examine how a new rail corridor might alter freight mode allocation and overall trade performance. The subsequent sections of the paper therefore, focus on how the China-Thailand Railway could alter the relative competitiveness of road, rail, and sea transport and, in turn, shape the future evolution of Thailand-Guangxi trade.

## 3. Methodology

### 3.1. Overall research design

This study adopts a mixed-methods design that combines expert interviews, the Analytic Hierarchy Process (AHP), a scenario-based multinomial Logit model, and a SWOT analysis. The objective is to evaluate how the China-Thailand Railway is likely to affect Thailand’s trade with Guangxi by comparing alternative transport modes (road, sea, and rail) in terms of logistics performance and strategic implications.

The research proceeded in four main stages. First, semi-structured interviews with domain experts were conducted to identify key route-choice criteria, to obtain quantitative information on transport time and cost for different routes, and to collect qualitative views on the opportunities and challenges associated with the China-Thailand Railway. Second, AHP was applied to the expert judgements to derive the relative importance (weights) of six route-choice criteria. Third, these weights and the attribute values of each transport mode were used to construct a generalized cost index and a scenario-based multinomial Logit model to estimate mode-choice probabilities. Finally, the quantitative findings and the qualitative interview evidence were integrated into a SWOT framework to discuss strategic implications for Thailand-Guangxi trade.

**Fig 1:**
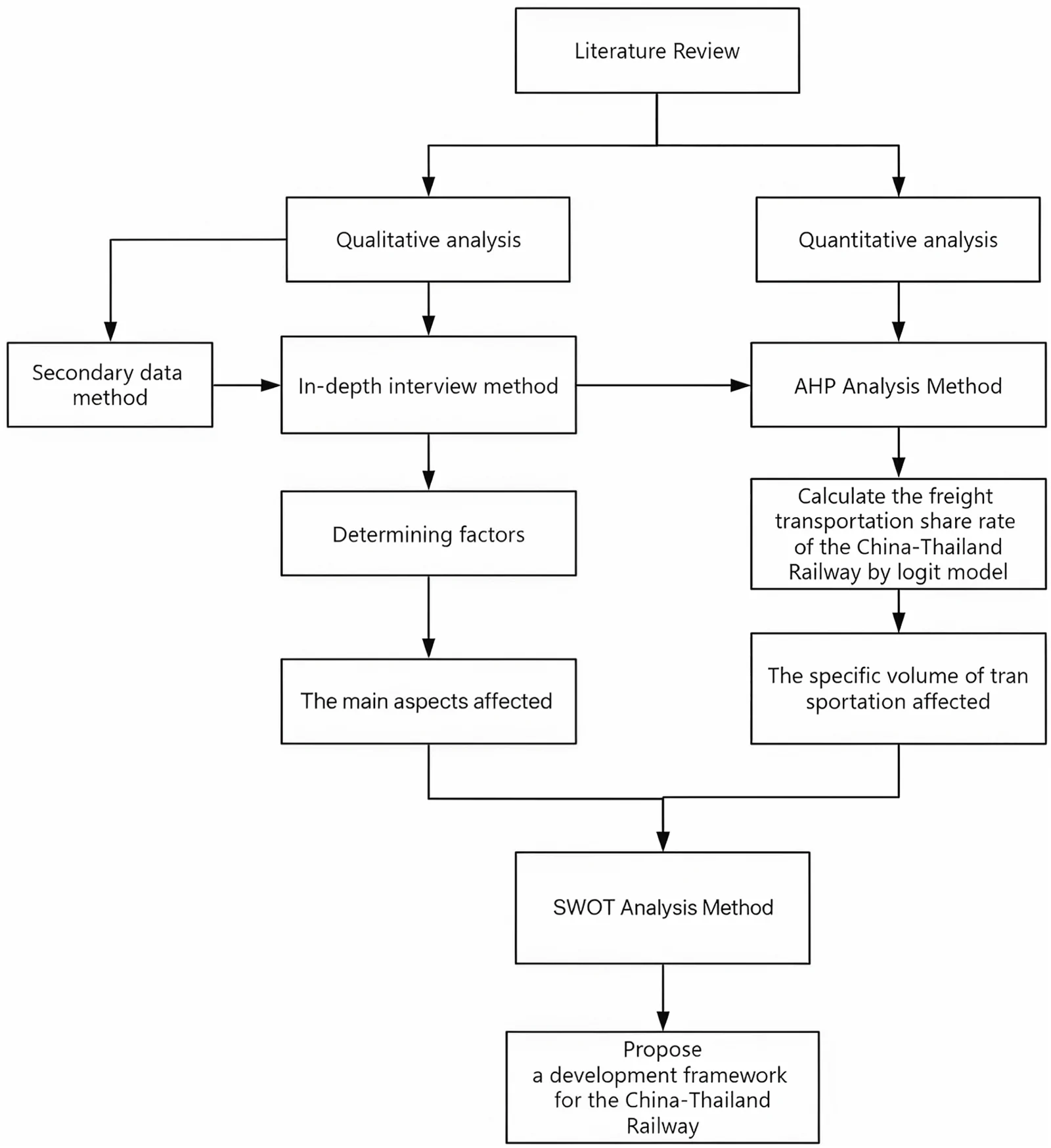
Research framework and analytical flow of the study.

Fig 1 summarizes the overall research framework and the sequence of qualitative and quantitative steps in this study. Starting from the literature review and secondary data analysis, the process moves through expert interviews, determination of the key route-choice criteria, AHP-based weighting and scenario- based Logit modelling of transport mode shares, and finally to the SWOT analysis and the development framework proposed for the China-Thailand Railway.

### 3.2. Expert sampling and interview design

#### 3.2.1. Expert selection.

Eight experts were deliberately selected to cover the key institutional and logistical perspectives involved in Thailand-China trade and the construction and operation of cross-border transport corridors. The panel consists of:

- two customs officers from border customs offices(Pingxiang Customs of the People’s Republic of China and Yongzhou Customs of the People’s Republic of China)handling Thailand-China trade flows;
- two academics specializing in transport, logistics, and regional economic development (One scholar is affiliated with Yunnan University (China), and the other with Naresuan University (Thailand);
- three industry practitioners from logistics companies and fruit and rice trading firms that regularly move goods between Thailand and China (among the participating entities, the fruit company is headquartered in Kunming, China; the rice company, in Nanning, China; and the freight forwarding and transportation company, in Bangkok, Thailand.);
- one senior engineer from a railway construction enterprise involved in the China-Laos and China- Thailand railway projects. These experts were chosen because they have direct operational or analytical experience with existing routes (e.g., R3A, R9, sea routes, China-Laos railway) and are familiar with anticipated conditions on the China-Thailand Railway. The composition of the panel ensures that both policy/regulatory and business/logistics perspectives are represented.

#### 3.2.2. Interview procedure.

Semi-structured interviews were conducted individually with each expert. The interview guide covered four main topics:

1. the expert’s role and experience with Thailand-China trade and cross-border transport;
2. perceptions of how the China-Thailand Railway may affect trade between Thailand and Guangxi (e.g., changes in cost, time, reliability, and security);
3. factual information on existing and planned transport routes, including approximate door-to-door transport time, freight rates (yuan/ton·km), and key operational characteristics for road, sea, and rail options; and
4. an AHP questionnaire in which experts compared six route-choice criteria in a pairwise fashion.

Interviews were conducted face-to-face or online, depending on availability, and typically lasted 30–60 minutes. With consent, detailed notes were taken. The qualitative responses were later organized into themes such as logistics cost, transit time, customs procedures, smuggling and security, and infrastructure capacity. These themes were used both to support the AHP and Logit modelling and to populate the SWOT analysis.

Ethical considerations were respected by informing all participants about the purpose of the study, guaranteeing anonymity in the reporting, and allowing experts to review and clarify key factual information where necessary.

Informed consent confirmation: All eight participating experts provided full informed consent prior to conducting semi-structured interviews. Each participant received a pre-interview information sheet that clearly stated the research purpose, data usage rules, publication plan, anonymity protection measures, and their right to withdraw participation at any time without penalty.

Type of consent obtained: Verbal informed consent was collected from all experts.

Documentation method: The researcher recorded each participant’s verbal consent statement in standardized interview logs, with detailed time stamps; all audio recordings (collected with prior permission) captured verbal consent confirmation at the opening of each interview as documentary evidence.

Witness requirement: A second research team member acted as an independent witness for each interview, signing the interview log to validate verbal consent records.

No minor participants: All interviewed experts are adult industry practitioners, customs officials, academic researchers, and railway engineering professionals aged over 18; no minors were involved in this research, so parental/guardian consent was not required.

Anonymization measures: All expert personal identifiers (full names, work unit precise addresses, contact information) were permanently removed from all raw data, tables, appendices, and manuscript text. Experts are only labelled as E1–E8 in all analytical materials, with no traceable personal information retained in published content or shared datasets.

### 3.3. AHP modelling of route-choice criteria

#### 3.3.1. Criteria definition.

Based on a review of the route-choice and freight transport literature and on preliminary discussions with the experts, six criteria were identified as the main factors affecting shippers’ choice among alternative transport modes: The Analytic Hierarchy Process (AHP) is used to quantify the relative importance of the main operational factors influencing shippers’ mode choice among road, rail, and sea transport. The factors and their representations are given in Table 3. These variables provide a common framework forthe AHP weighting and the subsequent mode-share analysis.

**Table 3:** Principles for determining influencing factors.

Influencing factor	Representative symbol	Definition
Transportation time	C1	Total transportation time of the i-th mode of transportation, from origin to destination, including border processes
Transportation cost	C2	Freight cost per ton for the i-th mode of transportation
Convenience	C3	Overall assessment of customs procedures, documentation requirements, and road and route conditions, scored by experts
Safety	C4	Safety level of the i-th mode, reflected inversely by the cargo damage or loss rate
Punctuality	C5	Punctuality rate ofthe i-th mode of transportation
Transportation capacity	C6	Maximum load (weight when fully loaded) for the i-th mode of transportation

#### 3.3.2. Individual expert judgement and consistency (moved from section 4.2 to methodology).

Each of the eight experts completed a 6 × 6 pairwise comparison matrix for the six influencing factors. The matrices were processed using the standard AHP procedure. All experts provided logically ordered judgments with acceptable consistency. Although weight profiles vary somewhat across experts due to their different institutional backgrounds in customs administrations, academia, logistics enterprises, trading companies, and railway construction, a stable pattern emerges. Across experts, transportation time and transportation cost are consistently ranked highest. Convenience, safety, punctuality, and transportation capacity are regarded as relevant but secondary. Sensitivity checks, based on temporarily excluding individual experts from the Aggregation, indicate that the combined weights are robust and that the ranking of factors remains unchanged. All eight experts’ judgements are therefore retained in the computation of the final weights.

#### 3.3.3. Pairwise comparisons and individual weights.

For each expert, a 6 × 6 pairwise comparison matrix was constructed using the standard Saaty1–9 scale. In each matrix, element a_ij represents_ the relative importance of criterion i over criterion j, where values of 1, 3, 5, 7 and 9 denote equal, slightly, clearly, strongly, and significantly greater importance, respectively, and the even numbers act as intermediate intensities. Reciprocal values (1/3, 1/5, …, 1/9) indicate the corresponding degrees of lesser importance, and the diagonal elements a_ij equal_ 1. For each expert, the weight vector w ^(e) for^ the six criteria were obtained using the geometric-mean method. For expert e and criterion i:


$$\begin{array}{c} g_{i}^{(e)}={\left(\prod_{j=\text{1}}^{\text{6}}a_{ij}^{(e)}\right)}^{\text{1}/\text{6}},w_{i}^{(e)}=\frac{g_{i}^{(e)}}{\sum_{k=\text{1}}^{\text{6}}g_{k}^{(e)}} \end{array}$$ (1)

To assess internal consistency, the principal eigenvalue λ_m_^(e^_a_^)^_x_ approximated by:


$$\begin{array}{c} \lambda_{max}^{(e)}=\frac{\text{1}}{\text{6}}\sum_{i=\text{1}}^{\text{6}}\frac{{(A^{\left(e\right)}w^{\left(e\right)})}_{i}}{w_{i}^{(e)}} \end{array}$$ (2)

where the consistency index (CI) and consistency ratio (CR) were computed as:


$$\begin{array}{c} {\;CI}^{(e)}=\frac{\lambda_{max}^{(e)}-\text{6}}{\text{6}-\text{1}},{CR}^{(e)}=\frac{{CI}^{(e)}}{RI} \end{array}$$ (3)

Where RI = 1.24 is the random index for matrices of order six. Following common AHP practice, a judgment set is considered acceptable if CR^(e) ≤^ 0.10. All eight experts satisfied this criterion, so no expert was excluded on the basis of consistency.

#### 3.3.4. Aggregation of expert judgements.

To obtain group weights that reflect the combined opinion of all experts, the individual weight vectors were aggregated criterion-wise using the geometric mean:


$$\begin{array}{c} {\tilde{w}}_{i}={\left(\prod_{e=\text{1}}^{\text{8}}w_{i}^{(e)}\right)}^{\text{1}/\text{8}},w_{i}^{final}=\frac{{\tilde{w}}_{i}}{\sum_{k=\text{1}}^{\text{6}}{\tilde{w}}_{k}} \end{array}$$ (4)

This yields a single final weight vector w ^final^ for the six criteria, which is reported in the results section. As anticipated from the interview evidence, transport cost and transport time emerge as the two most important criteria. The detailed matrices, individual weights and consistency ratios are provided in an appendix and in an accompanying spreadsheet for transparency.

### 3.4. Construction of transport-mode scenarios

#### 3.4.1. Modes and routes considered.

The analysis focuses on three representative transport modes/routes for trade between Thailand and Guangxi:

1. Road transport, mainly via the R3A and R9 international highway corridors linking Bangkok with Kunming and then Guangxi;
2. Rail transport, via the planned China-Thailand Railway connected through the China-Laos Railway to Kunming and onward to Guangxi; and
3. Sea transport, via ocean shipping from Thai ports (e.g., Bangkok or Laem Chabang) to Chinese seaports (e.g., Guangzhou) and then inland connections to Guangxi.

These modes capture the main current and prospective alternatives for shippers in the study context.

#### 3.4.2. Attribute values and normalization.

For each mode i, attribute values x_ik were_ assigned for the six criteria. Where available, numerical information on transit times and freight rates (yuan/ton·km) was obtained from the interviews. For example, experts reported R3A/R9 road transport taking about 40–46 hours at roughly 0.9–1.1 yuan/ton·km, sea transport around 130 hours at about 0.04 yuan/ton·km, and the future China-Thailand rail scenario around 35–40 hours at about 0.2 yuan/ton·km. Qualitative assessments of convenience, safety, punctuality, and capacity were converted into numerical scores on a consistent 0–10 scale, with higher values indicating more favorable performance.

Because the criteria have different units and directions (e.g., lower cost and time are preferred, while higher safety and capacity are preferred), all attributes were normalized before combination. For “cost- type” criteria (time, cost, and convenience), values were normalized so that higher values correspond to better performance (e.g., by dividing the minimum observed value by each mode’s value). For “benefit- type” criteria (safety, punctuality, capacity), normalization was performed by dividing each value by the maximum observed value. This ensures that all normalized attributes lie between 0 and 1 and are comparable across criteria.

### 3.5. Generalized cost index and Logit model

#### 3.5.1. Develop a standardized single-indicator scoring framework.

Differentiate between cost-oriented and benefit-oriented indicators, and apply two distinct standardization formulas to harmonize measurement units and align directional interpretations (i.e., higher scores consistently reflect superior performance):

Cost-oriented indicators (Cost C_1_, Time C_2_):


$$\begin{array}{c} X_{ik}^{*}=\frac{Min(X_{K})}{X_{ik}} \end{array}$$ (5)

Benefit-oriented indicators (convenience C_3_, safety C_4_, punctuality C_5_, transportation capacity C_6_)


$$\begin{array}{c} X_{ik}^{*}=\frac{(X_{K})}{maxX_{ik}} \end{array}$$ (6)

#### 3.5.2. Generalized cost index.

Using the final AHP weights w ^final^, a generalized cost (GC) index was computed for each mode i as a weighted sum of the normalised attributes:


$$\begin{array}{c} {GC}_{i}=\sum_{k=\text{1}}^{\text{6}}w_{K}^{final}X_{ik}^{*} \end{array}$$ (7)

wherex*_ik_ denotes the normalized value of criterion k for mode i. Because higher values of x*_ik_ represent better performance after transformation, a higher Gc_i_ indicates a more attractive mode.

#### 3.5.3. Multinomial Logit specification.

To translate these generalized cost scores into relative choice probabilities, a multinomial Logit model was specified. The systematic utility of mode i is defined as


$$\begin{array}{c} u_{i}={aGC}_{i} \end{array}$$ (8)

Where (α > 0) is a scaling parameter (set to 1 for convenience, since only relative differences matter for probabilities). The probability that a representative shipper chooses mode i is then given by


$$\begin{array}{c} P_{i}=\frac{\text{exp}(u_{i})}{\sum_{j}\text{exp}(u_{j})} \end{array}$$ (9)

This formulation ensures that each p_i_ lies between 0 and 1, and that the probabilities sum to one across modes. Because the model uses expert-informed scenario data rather than survey observations of individual shippers, it should be interpreted as a scenario-based mode-choice simulation rather than an econometric ally estimated behavioral model. Nevertheless, it provides a transparent and internally consistent way to compare the relative attractiveness of the different transport modes under plausible assumptions about the China-Thailand Railway.

### 3.6. SWOT analysis

The final step integrates the quantitative and qualitative evidence into a SWOT framework to discuss the strategic implications of the China-Thailand Railway for Thailand-Guangxi trade. Strengths and weaknesses were derived mainly from the railway’s relative performance in terms of cost, time, capacity, safety, and reliability compared with road and sea, as captured by the AHP weights and the Logit results.

Opportunities and threats were identified from the interview narratives, including regional economic development along the corridor, potential diversion of trade from other Chinese provinces, environmental and security concerns, and institutional challenges in customs and border management.

By linking each SWOT element to either quantified indicators (generalized cost and mode probabilities) or explicit expert statements, the analysis avoids purely speculative judgements. It ensures that the strategic discussion is grounded in the mixed-methods evidence generated in the previous steps. At the same time, it should be noted that the findings are based on a relatively small expert sample and scenario- based assumptions regarding the China-Thailand Railway; the results therefore provide informed projections rather than precise forecasts and should be interpreted with these limitations in mind.

## 4. Results (Strictly empirical outputs only; all interpretive discussion moved to separate section 5)

### 4.1. Interview findings and key factors

The main objective of this study is to evaluate how the China-Thailand Railway will influence Thailand’s trade with Guangxi, particularly through changes in freight routes and logistics performance. The eight expert interviews provide the first layer of evidence by clarifying which development dimensions are perceived as most affected and which factors drive shippers’ transport mode choices.

On the macro level, experts were asked to rank the importance of five broad impact dimensions identified in the literature: economy and trade, logistics, tourism, environment, and security. As presented in Table 4, all eight experts consistently rated economy and trade as the most influential dimension, followed by logistics. Tourism and environmental impacts were regarded as secondary concerns, while security received the lowest priority ranking across all participants.

**Table 4:** Selection of the importance of influencing factors by eight experts.

Expert/ Factor	Economy and trade	Logistics	Tourism	Environment	Security
1	1	2	4	3	5
2	1	3	2	4	5
3	2	1	3	4	5
4	1	2	3	5	4
5	2	1	3	4	5
6	1	2	3	5	4
7	1	2	3	4	5
8	1	3	2	4	5

(Note: “1 “indicates the highest importance, “5 “the lowest, for each expert.)

At the micro level, the interviews confirm that shippers’ route choices between road, rail, and sea are mainly driven by six operational criteria: transport time, transport cost, convenience, safety, punctuality, and transport capacity. Experts emphasized that transport cost and transport time are especially critical for long-distance trade in agricultural and manufactured products, whereas convenience (customs procedures, documentation, and transshipment), safety (risk of damage or loss), punctuality (on-time performance), and capacity (ability to handle large and growing volumes) also influence actual decisions. These six criteria are exactly those used in the AHP model and subsequently in the logit-based mode-choice analysis, ensuring that the quantitative modelling is grounded in the experts’ practical experience.

Taken together, the interview findings provide a coherent qualitative foundation for the subsequent AHP and Logit results: they confirm the macro-impact dimensions used in the SWOT analysis and support the choice of criteria and modes in the quantitative part of the study.

### 4.2. AHP results for route-choice criteria

#### 4.2.1. Final combined weights and implications.

**Fig 2:**
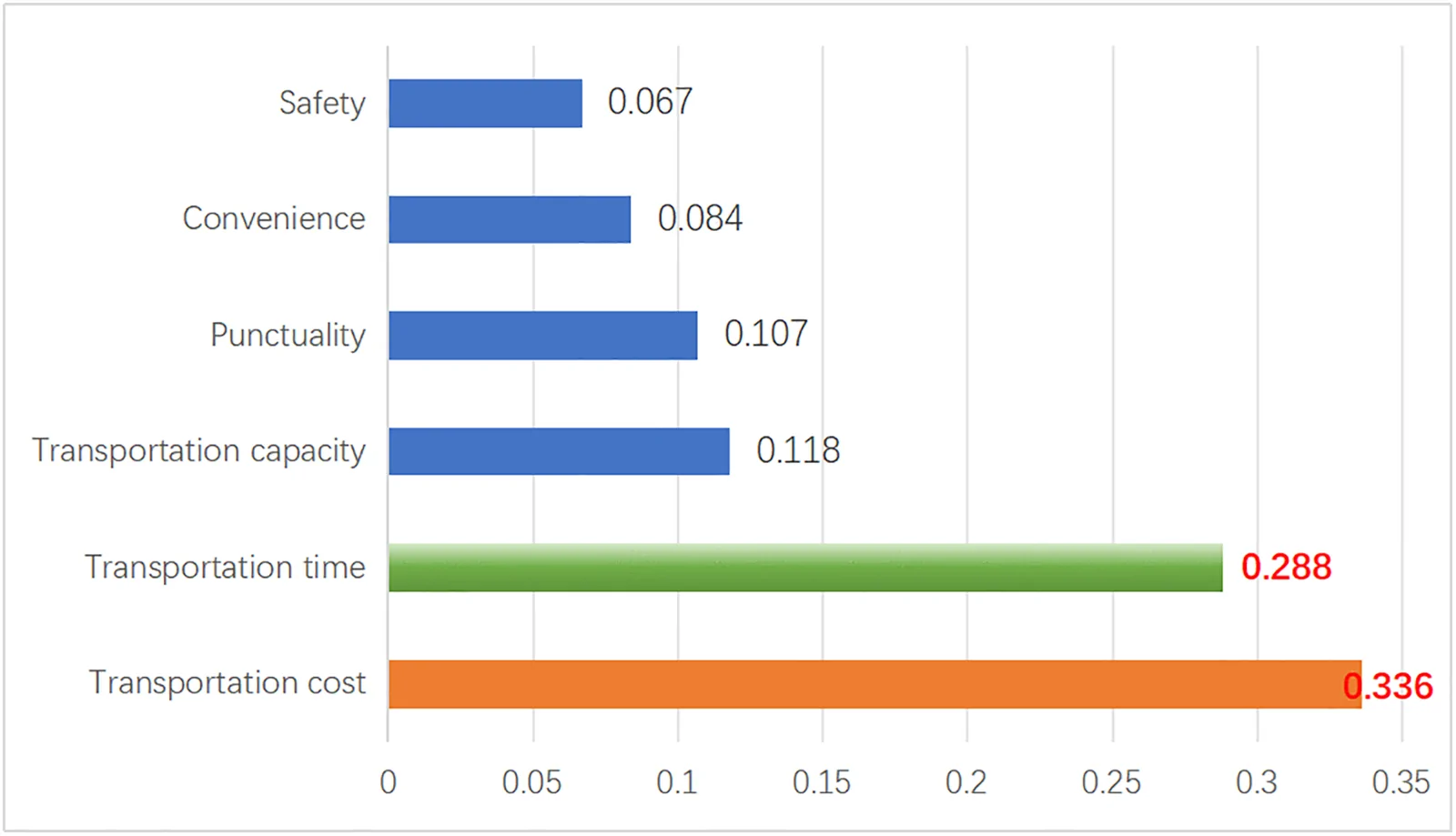
The weight of influencing factors by AHP Weight.

Individual weight vectors were aggregated by applying the geometric mean across experts for each factor, followed by normalization to ensure that the weights sum to one. The resulting combined weights are reported in Fig 2.

Transportation cost and transportation time together account for approximately 62% of the total weight, confirming their dominant role in freight mode choice in the Thailand-Guangxi corridor. Transportation capacity and punctuality rank second in importance, reflecting concerns about each mode’s ability to handle increasing freight volumes reliably and on schedule. Convenience and safety have lower, but still meaningful, weights and capture the influence of procedural complexity and cargo risk on shippers’ decisions. For the China-Thailand Railway, the combined weights underscore the central importance of competitive freight tariffs and short, reliable transit times. Sufficient capacity and high punctuality further enhance the attractiveness of rail services, while improvements in convenience and safety provide additional benefits. The weight structure, therefore, indicates that the railway’s competitive position depends primarily on its cost-time performance, supported by robust capacity and operational reliability.

### 4.3. Logit-based prediction of transport mode shares

The relative competitiveness of road, railway, and sea transport between Thailand and Guangxi is assessed using a multinomial Logit model that combines AHP-derived weights with calibrated attributes for each mode. The model evaluates how differences in transportation time, cost, convenience, safety, punctuality, and capacity translate into expected market shares for each transport option.

#### 4.3.1. Attribute values for each mode of transportation.

The influencing factors and their representative symbols have been defined in Table 3. Based on distance, tariff, and performance information provided by the experts, parameter values are assigned to each mode. These values are summarized in Table 5. Road transport between Thailand and Guangxi offers relatively short transit times and high flexibility, but is characterized by higher freight costs per ton and limited capacity per vehicle. Railway transport via the China-Thailand Railway, connected to the China-Laos Railway, offers shorter transit times than sea transport, lower costs than long-distance road transport, and substantially higher capacity per train. Sea transport retains a strong advantage in terms of freight rates and very large capacity, but requires significantly longer transit times. At the final stage of data determination, the median was selected as the representative measure for each data set.

**Table 5:** Parameter values of the influencing factors for each mode of transportation.

Mode of transportation	Time (hours)	Cost (yuan/ton·km)	Convenience (score 0–10)	Safety (%)	Punctuality (%)	Transportation capacity (ton)
Road transportation	45	0.90	9	96	75	50
Railway transportation	37	0.20	6	98	97	20,000
Sea transportation	130	0.04	8	92	60	100,000

These calibrated values capture the trade-offs facing shippers. Road transport is faster than sea but more expensive and capacity-constrained. Railway transport combines a relatively short time with moderate cost and large capacity. Sea transport is slow but offers the lowest freight cost and very high capacity. Normalization must be applied to all six evaluation indicators. Sea transport is slow but offers the lowest freight cost and very high capacity. negative indicators (transport time, transport cost) and positive indicators (convenience, safety, punctuality, transport capacity). Second, we supplemented unified and standardized normalization formulas for two types of indicators: negative indicators adopt minimum value/ original attribute value normalization, and positive indicators adopt original attribute value/ maximum value normalization, ensuring all normalized values range from 0 to 1 with consistent evaluation direction (higher value represents better performance).

**Fig 3:**
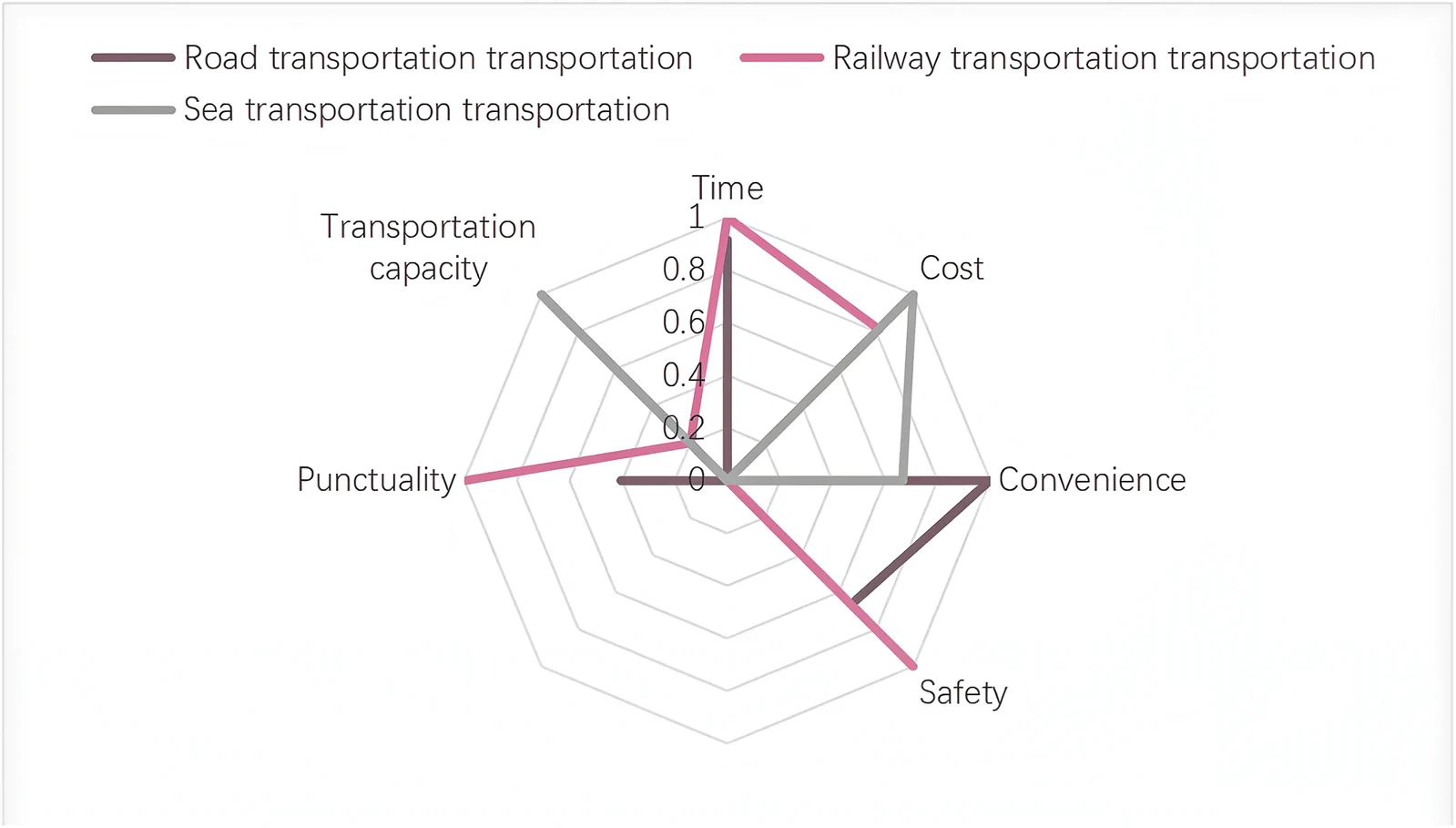
The performance of various transportation modes under differing factors.

In order to facilitate a more thorough analysis of the data, the author is considering converting the table into a radar chart, Therefore, the author normalized the data. Subsequently, a radar chart is constructed using the processed data in Fig 3. We standardized raw attribute parameters for three transport modes via differentiated normalization formulas, and the unified 0–1 normalized performance values are fully displayed in Table 6.

**Table 6:** Data normalization processing.

Factors	Road transportation	Railway transportation	Sea transportation
Time	0.914	1.000	0.000
Cost	0.000	0.814	1.000
Convenience	1.000	0.000	0.667
Safety	0.667	1.000	0.000
Punctuality	0.405	1.000	0.000
Transportation capacity	0.000	0.200	1.000

#### 4.3.2. Predicted sharing rates of transport modes.

**Fig 4:**
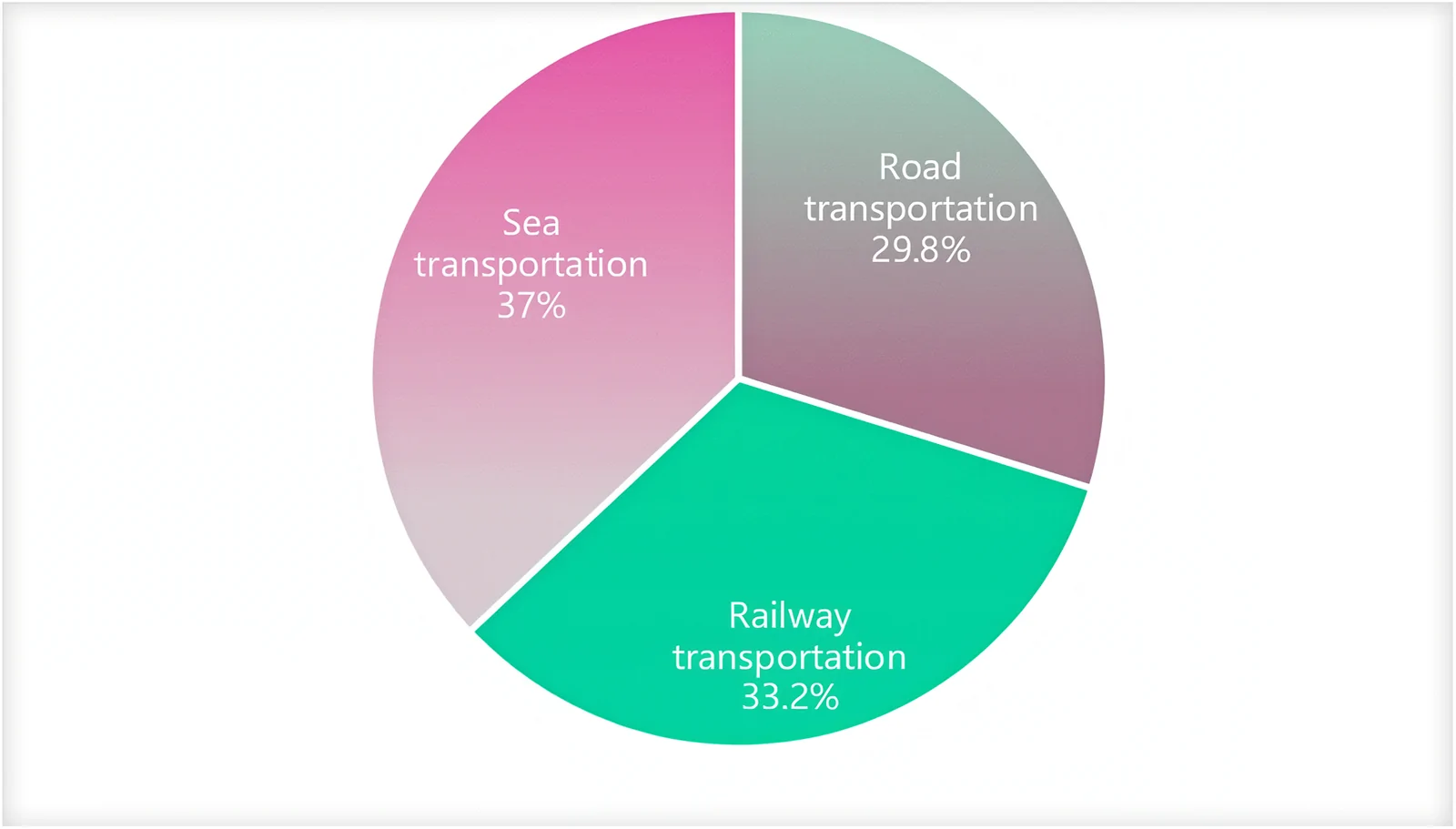
Share ratio of transport modes.

The parameter values in Table 6 are normalized and combined with the AHP weights to obtain a composite performance score for each mode. These scores are then entered into the multinomial Logit model to derive the predicted sharing rates of road, railway, and sea transport in the study corridor. The resulting estimates are reported in Fig 4.

The estimates indicate that, under the assumed scenario, sea transport remains the most widely used mode, with a predicted share of approximately 37.0%. This reflects the strong cost and capacity advantages of maritime shipping, despite its longer transit time. The China-Thailand Railway is estimated to have a share of about 33.2%, supported by a favourable balance of cost, time, capacity, and punctuality. Road transport accounts for around 29.8% of the predicted freight volume, constrained by higher unit costs and limited vehicle capacity, despite its comparatively short transit time.

Theoretical rationalization: The scaling parameter α only regulates the dispersion degree of exponential utility values and will never reverse the relative ranking of generalized competitiveness among transport modes. Setting α = 1 provides intuitive linear mapping between composite generalized cost scores and choice utilities for baseline comparison.

Tested values: We conduct simulations with α = 0.5, α = 1(baseline), and α = 2.

Robust finding: Even with extreme adjustments of α, the market share of each transport mode fluctuates within ±5 percentage points, and the fixed competitive hierarchy (sea > railway > road) remains unchanged, verifying the stability of our core ranking conclusion. Across all extreme boundary parameter combinations, the order of modal market share never reverses, and the fluctuation bandwidth of each share is controlled within 3.3 percentage points in Table 7.

**Table 7:** Comprehensive bias comparison table.

Scaling α	Road Share	Rail Share	Sea Share	Road Absolute Bias (pct)	Rail Absolute Bias (pct)	Sea Absolute Bias (pct)	Bias Type Classification
0.5	31.3%	33.2%	35.5%	+1.5	0.0	−1.5	Low-α under-sensitivity bias
1.0 (Baseline)	29.8%	33.2%	37.0%	0.0	0.0	0.0	Zero bias (calibrated benchmark)
2.0	25.4%	32.5%	42.2%	−4.4	−0.7	+5.2	High-α over-sensitivity bias

## 5. Separate discussion section (newly added per reviewer request; all interpretive analysis moved here)

### 5.1. Interpretation of AHP weight results

Transportation cost and transportation time together account for approximately 62% of the total weight, confirming their dominant role in freight mode choice in the Thailand-Guangxi corridor. Transportation capacity and punctuality rank second in importance, reflecting concerns about each mode’s ability to handle increasing freight volumes reliably and on schedule. Convenience and safety have lower, but still meaningful, weights and capture the influence of procedural complexity and cargo risk on shippers’ decisions. For the China-Thailand Railway, the combined weights underscore the central importance of competitive freight tariffs and short, reliable transit times. Sufficient capacity and high punctuality further enhance the attractiveness of rail services, while improvements in convenience and safety provide additional benefits. The weight structure, therefore, indicates that the railway’s competitive position depends primarily on its cost-time performance, supported by robust capacity and operational reliability.

### 5.2. Analysis of simulated transport mode market shares

The multinomial Logit scenario simulation predicts ocean shipping will retain the largest single market share (37.0%) after full China-Thailand Railway operation, driven by its ultra-low unit freight cost and massive single-vessel load capacity. Maritime transport remains the optimal choice for low-value, non-time-sensitive bulk goods such as raw rubber and industrial chemical raw materials, where long transit durations create minimal economic losses.

The China-Thailand Railway achieves a projected 33.2% freight market share, ranking as the second-most competitive logistics channel. The rail corridor strikes an optimal balance between transit speed (far faster than sea transport) and unit cost (far cheaper than long-distance highway trucking), making it uniquely suited for high-value time-sensitive commodities including fresh tropical fruit, electronic components and automotive spare parts. The simulation verifies the railway’s capacity to become a core competitor displacing volumes from both road and sea transport, fulfilling the policy goal of diversifying Sino-Thai cross-border logistics channels.

Road highway transport captures the smallest projected share at 29.8%, constrained by its high per-ton-kilometer freight cost and limited single-vehicle capacity despite its moderately fast door-to-door delivery time. Highway transport will remain viable only for small-batch, emergency urgent shipments with extremely high time priority, rather than large-scale bulk trade flows.

### 5.3. SWOT analysis and development strategies

A SWOT analysis is conducted to integrate qualitative evidence from expert interviews with quantitative findings from the AHP and Logit models, and to assess the strategic implications of the China-Thailand Railway for Thailand-Guangxi trade. The main strengths, weaknesses, opportunities, and threats identified in the analysis are summarized in Table 8.

**Table 8:** SWOT analysis of the impact of the China-Thailand Railway project.

S (Strengths)	W (Weaknesses)	O (Opportunities)	T (Threats)
1.Reduces logistics costs for Thailand-Guangxi import and export trade	1. Supporting infrastructure and local feeder networks are incomplete along parts of the corridor.	1. Economic growth along the corridor increases demand for higher-quality and more efficient logistics services.	1. Mature maritime routes and alternative land corridors create strong competition for freight flows.
2.Shortens transit time compared with sea transport and provides reliable, all- weather services.	2. Limited collection and distribution systems constrain door-to-door services for small and fragmented shipments.	2. Regional integration and Belt and Road policies support deeper trade facilitation and more integrated value chains.	2. Financial and political risks related to large-scale infrastructure may affect long-term project stability.
3.Offers large transport capacity and supports a modal shift from road to rail, reducing emissions.	3. Information, digital, and service systems lag behind the requirements of modern multi modal and integrated logistics.	3. Growing demand or digital, information-based, and green logistics encourages upgrading of technologies, management systems, and service models.	3. Increased customs and inspection workload raises the difficulty of risk control, including smuggling, illegal trade, and cross-border fraud.
4. Facilitates the development of inland logistics hubs and trade clusters around key nodes along the route.	4. Coordination among customs, inspection, and railway operating agencies is not yet fully harmonized, reducing operational efficiency.	4. The corridor can support tourism and cultural exchange, including the development of tourist and “humanistic” train products linked to trade promotion.	4. Underestimation of capacity is possible if supporting infrastructure and demand coordination do not keep pace with mainline construction.
5. Enhances the openness of border regions and strengthens the external connectivity of Guangxi and adjacent areas.	5. Local human resources in logistics, supply chain management, and e commerce are insufficient in some regions along the route.	5. Policy support for low-carbon transport and inland opening provides a favorable environment for innovative rail-based logistics products and services to emerge.	5. External shocks such as global economic downturns, pandemics, or geopolitical tensions may disrupt cross-border trade flows and reduce demand for rail services.

#### 5.3.1. SO developmental strategy.

The strengths identified in the SWOT analysis include lower logistics costs, shorter transit times than sea transport, high capacity, all-weather operation, and environmental advantages compared with road transport. These features are consistent with the AHP results, which assign the highest weights to transportation cost and time, and with the Logit-based estimates, which indicate that railway transport can capture a substantial share of freight under plausible operating conditions.

The SO strategy focuses on using these strengths to exploit favorable external conditions. Rising demand for high-value agricultural and manufactured exports, continuous economic growth along the corridor, and policy support for regional integration and low-carbon transport create a conducive environment for rail-based trade expansion. Priority measures include promoting the China-Thailand Railway as the preferred mode for time-sensitive and higher-value goods, developing logistics and value-added service clusters at key nodes, and encouraging multimodal transport solutions in which rail provides the backbone of regional freight movements.

#### 5.3.2. ST innovative diversification strategy.

Several external threats emerge from the SWOT analysis, including strong competition from well- established sea routes and potential rival land corridors, as well as financial and political risks. The ST strategy draws on the strengths of the railway, particularly its reliability, security, capacity, and environmental performance, to develop innovative service offerings that differentiate rail from competing modes.

Examples include specialized block trains for agricultural products, services tailored to e-commerce and express freight, and tourist-oriented or “humanistic” trains that link cultural and tourism resources along the route with trade flows. Flexible tariff structures and service packages can be designed to maintain the attractiveness of rail-based solutions even when sea freight prices fluctuate or competing infrastructure projects advance. In this way, the strengths of the China-Thailand Railway are used to mitigate external threats and to consolidate its position within the regional logistics system.

#### 5.3.3. WO turning-the-tide strategy.

The SWOT analysis also reveals internal weaknesses, notably incomplete supporting infrastructure, gaps in digital and information systems, limited local human resources in logistics and e-commerce, and the need for closer institutional coordination among border management agencies. At the same time, there are substantial opportunities created by national and regional development strategies, trade facilitation initiatives, and the potential for freight modal shift from road to rail. The WO strategy aims to address these weaknesses to take advantage of available opportunities. Key actions include accelerating the construction and upgrading of logistics parks, dry ports, and feeder infrastructure; improving digital platforms that provide shippers with transparent information on schedules, tariffs, and service quality; implementing training programmed to strengthen local logistics and e-commerce capabilities; and deepening cooperation among Thai, Lao, and Chinese authorities to streamline customs and inspection procedures. Successfully addressing these weaknesses will enhance the effective use of the China-Thailand Railway and strengthen its contribution to regional development.

#### 5.3.4. WT defensive strategy.

If internal weaknesses are not resolved and external threats intensify, the benefits of the China-Thailand Railway may fall short of expectations. The WT strategy focuses on defensive measures designed to safeguard trade flows and limit downside risks while the project and its supporting systems mature.

These measures include maintaining redundancy and flexibility in regional logistics networks, monitoring developments in competing corridors and sea freight markets, and preparing contingency plans for possible operational, financial, or political disruptions. Strengthening institutional cooperation mechanisms and establishing channels for timely information sharing and joint problem-solving among relevant authorities can further reduce vulnerability to adverse shocks. Such defensive arrangements help to ensure that Thailand-Guangxi trade remains resilient while the more proactive SO, ST, and WO strategies are implemented and the full potential of the China-Thailand Railway is gradually released.

### 5.4. Full statement of research limitations (moved from original conclusion per reviewer request)

This research carries several notable methodological and sample limitations that constrain generalizability of the findings:

1. Scenario hypothetical parameters: All rail transport cost, transit time and capacity indicators are forward-looking projections for the uncompleted China-Thailand Railway Phase II (target completion 2030–2031), rather than real-world operational data. Actual market shares may deviate significantly if final railway tariffs, transit speeds or gauge transshipment efficiency differ from expert-calibrated assumptions;
2. Small non-probabilistic expert sample: Purposive sampling of eight industry experts cannot represent the full population of cross-border logistics stakeholders; follow-up research should adopt large-scale random shipper surveys to validate AHP weight results;
3. Static single-period simulation: The multinomial Logit model delivers a static long-run market share projection without incorporating dynamic factors such as fluctuating global commodity prices, seasonal agricultural export peaks or policy tariff adjustments; future extensions can integrate dynamic panel trade data to build time-varying mode choice models.

## 6. Conclusion (condensed per reviewer request; limitations removed to section 5)

This study examined the likely impact of the China-Thailand Railway on Thailand’s trade with Guangxi by analyzing how the project changes the relative attractiveness of road, rail, and sea transport and by assessing the broader strategic implications for regional development. A mixed-methods framework was applied, combining a review of the relevant literature and secondary data with expert interviews, an Analytic Hierarchy Process (AHP) evaluation of route-choice criteria, a multinomial Logit model of transport mode choice, and a SWOT analysis. This design links qualitative insights on trade and logistics dynamics with quantitative estimates of mode shares and a structured strategic assessment.

The AHP results show that transportation cost and transportation time are the dominant determinants of freight mode choice in the Thailand-Guangxi corridor, together accounting for about 62% of the total weight. Transportation capacity and punctuality rank second in importance, while convenience and safety have lower but still meaningful weights. These findings are consistent with expert perceptions that cost-time performance and the ability to move large volumes reliably are critical for long-distance trade in agricultural and manufactured products. For the China-Thailand Railway, the weight structure shows that competitive freight tariffs and short, reliable transit times are essential to attracting freight away from existing road and sea routes.

Using the AHP weights together with calibrated attribute values for road, rail, and sea transport, the Logit model estimates that, under a plausible operating scenario, sea transport would capture about 37.0% of freight, railway transport about 33.2% and road transport about 29.8%. Sea transport remains attractive because of very low freight rates and high capacity. However, the China-Thailand Railway emerges as a strong competitor, offering a favorable balance of cost, time, capacity, and punctuality. These results suggest that, provided actual tariffs and service performance are close to the assumed values, the project has the potential to capture a substantial share of existing freight flows and to induce a partial modal shift from road to rail, with positive implications for logistics efficiency and environmental performance.

The SWOT analysis, informed by both the interviews and the AHP-Logit results, indicates that the China- Thailand Railway possesses clear strengths in cost-time performance, capacity, reliability, and environmental characteristics, and that it benefits from opportunities created by regional economic growth, trade facilitation initiatives, and demand for digital and green logistics. At the same time, weaknesses remain in supporting infrastructure, information systems, human resources, and institutional coordination, while threats include competition from mature maritime routes and alternative land corridors, as well as financial, political, and macroeconomic risks. The resulting SO, ST, WO, and WT strategies emphases the need to capitalize on the railway’s strengths to promote trade and logistics upgrading, develop innovative and differentiated rail services, accelerate improvements to supporting systems, and maintain defensive arrangements that preserve resilience in the face of uncertainty.
