# Supplementary material for: The effect of the China-Thailand railway on Thailand’s trade with China Guangxi Province
Source: PLoS One. 2026 Sep 18;21(9):e0350972. doi: 10.1371/journal.pone.0350972 (PMC13588508; doi:10.1371/journal.pone.0350972)
Supplement: Supplementary file 1 [file pone.0350972.s001.docx]

# Comments to the Authors

| **Manuscript Number** | PONE-D-26-23847 |
| --- | --- |
| **Title** | The Effect of the China-Thailand Railway on Thailand's Trade to China Guangxi Province |
| **Journal** | PLOS ONE |

## A. Main Comments

1. Several statistics cited in the Introduction and Literature Review are not attributed to any source. For example, the first paragraph of the Introduction presents bilateral trade figures (e.g., China-Thailand trade of 887.6 billion yuan in 2023; Thai-Guangxi trade of 40.4 billion yuan) without a reference. The same issue recurs throughout the Literature Review: Table 1 (influencing factors across 30 studies) and Table 2 (Guangxi-ASEAN/Thailand trade volumes) are presented, but the surrounding narrative text largely stands alone, without citation to the listed studies or to a data source. Please clarify whether this content reflects the authors' own compilation/analysis or is drawn from the cited literature, and add the appropriate in-text citations throughout. In particular, please specify explicitly the source (database, agency, or publication) for the trade volume figures in Table 2.
2. Regarding the expert panel: please clarify the nationality/affiliation composition of the eight interviewed experts. As described, the panel appears to consist of customs officers, academics, logistics/trading practitioners, and a railway engineer, but it is not stated whether any Thai experts were included, or whether the panel was composed entirely of Chinese (Guangxi-based) experts. Given that the study explicitly examines a bilateral Thailand-China relationship, the absence of Thai-side expert perspectives (if that is the case) is an important limitation that should be stated clearly and justified, or addressed by including Thai experts in a revision.
3. The "Results and Discussion" section should contain only results; interpretive discussion currently embedded within it should be moved to a separate Discussion section so that readers can clearly distinguish empirical findings from the authors' interpretation. In addition, parts of Section 4.2 “AHP results for route-choice criteria” read as methodology rather than results, for instance, Table 4 (“Principles for determining influencing factors,” which defines the criteria and their symbols) and Section 4.2.1 (“Individual expert judgement and consistency,” which restates the AHP procedure) substantially duplicate content already presented in the Methodology section. Please move this material to Section 3 (Methodology) and keep Section 4 focused strictly on reporting outcomes (e.g., the final weights in Table 5, the predicted mode shares in Table 7).
4. The Conclusion is too long and includes content that belongs elsewhere, most notably the study's limitations. I recommend creating a separate Discussion section (following the restructured Results) that addresses interpretation, significance, and limitations of the study. The Conclusion should then be shortened to a concise summary of the main findings and their implications, so that a reader can grasp the essence of the study without re-reading the full limitations discussion.
5. The manuscript relies entirely on tables (eight tables, no figures) to present what is otherwise a substantial body of analytical work. I recommend adding data visualizations, for example, a bar chart of the AHP criteria weights (Table 5), a bar or pie chart of the predicted modal shares (Table 7), and/or a radar/spider chart comparing the three transport modes across the six criteria (Table 6) to improve the manuscript's readability and overall visual quality.

## B. Additional Comments

*The following observations supplement the points raised above.*

6. Data availability: the data availability statement should explicitly confirm where the AHP pairwise comparison matrices, individual expert weights, and consistency ratios (described in Section 3.3.3 as “provided in an appendix and in an accompanying spreadsheet”) will be deposited or made available, consistent with PLOS's data-sharing policy.
7. Internal inconsistency in the normalization of “Convenience” (Section 3.4.2): the text first states that convenience, safety, punctuality, and capacity are all scored on a 0–10 scale “with higher values indicating more favorable performance,” but then classifies convenience as a “cost-type” criterion requiring inverse normalization alongside time and cost (i.e., lower raw value = better), while safety/punctuality/capacity are treated as “benefit-type.” This is contradictory and should be clarified. Relatedly, Table 6 reports a Sea convenience score of 15, which exceeds the stated 0–10 scale — please correct this value or revise the scale description.
8. Precision of the Logit results / absence of sensitivity analysis: attribute values are reported as ranges in the text (e.g., road time “40–46 hours,” cost “0.9–1.1 yuan/ton·km”; rail “35–40 hours” at “about 0.2 yuan/ton·km”) but are collapsed into single deterministic point values in Table 6 with no stated rule for how the point value was chosen. In addition, the Logit scale parameter α is fixed at 1 “for convenience” with no justification or testing of alternative values. As a result, the headline estimates (37.0% / 33.2% / 29.8%) read as more precise than the underlying scenario assumptions support. A sensitivity analysis varying α and/or the attribute ranges (e.g., a low/base/high scenario) would substantially strengthen the credibility of the modal-share predictions and is recommended before publication.
9. Notation inconsistency: Section 3.3.1 labels the six criteria C1 (cost), C2 (time), etc., while Table 4 in Section 4.2 instead uses Ti (time), Ci (cost), Bi (convenience), Si (safety), Wi (punctuality), Zi (capacity). Please adopt a single, consistent symbol set and introduce it once, in the Methodology section.
10. Reference list: “Lauridsen, L. S. (2020)” and “Qin Lei. (2020)” are both listed with the identical title (“Drivers of China’s Regional Infrastructure Diplomacy: The Case of the Sino-Thai Railway Project,” Journal of Contemporary Asia, 50(3), 380–406). This appears to be a duplicated or misattributed reference and should be corrected. More generally, several DOI strings in the reference list contain garbled text (e.g., “carol%2520carroll%2520nki”), apparently an encoding artifact from a Chinese database export (CNKI); the full reference list should be checked and cleaned before resubmission.
11. Language and proofreading: the manuscript would benefit from a careful English-language proofreading pass. Examples noted during review: “multinational Logit specification” (heading of Section 3.5.2) should read “multinomial Logit specification”; “optimism mode allocation” (end of Introduction) appears to be a typo for “optimizing mode allocation”; “analyses” is used incorrectly as a present-tense verb in several places (e.g., “studies that analyses overseas infrastructure,” “Other studies analyses China-Laos Railway-induced changes…”) and should read “analyze/analyzes”; the boxes in Figure 1 contain typos (“SWOT Aanalysis Mehod,” “The main asoects affected”) that should read “SWOT Analysis Method” and “The main aspects affected.” The title/running text “Thailand's Trade to China Guangxi Province” also reads awkwardly and might be revised to “Thailand's Trade with Guangxi Province, China” or similar.

*End of comments.*
