# Supplementary material for: The effect of the China-Thailand railway on Thailand’s trade with China Guangxi Province
Source: PLoS One. 2026 Sep 18;21(9):e0350972. doi: 10.1371/journal.pone.0350972 (PMC13588508; doi:10.1371/journal.pone.0350972)
Supplement: Supplementary file 2 [file pone.0350972.s003.docx]

Dear Reviewer 1

We sincerely appreciate your rigorous, detailed, and constructive comments, which have greatly helped us improve the rigor, transparency, and academic standard of this study. We have carefully addressed every raised issue, revised the manuscript thoroughly, and clarified all ambiguous content. The point-by-point responses and corresponding revisions are presented as follows:

1. Several statistics cited in the Introduction and Literature Review are not attributed to any source. For example, the first paragraph of the Introduction presents bilateral trade figures (e.g., China-Thailand trade of 887.6 billion yuan in 2023; Thai-Guangxi trade of 40.4 billion yuan) without a reference. The same issue recurs throughout the Literature Review: Table 1 (influencing factors across 30 studies) and Table 2 (Guangxi-ASEAN/Thailand trade volumes) are presented, but the surrounding narrative text largely stands alone, without citation to the listed studies or to a data source. Please clarify whether this content reflects the authors' own compilation/analysis or is drawn from the cited literature, and add the appropriate in-text citations throughout. In particular, please specify explicitly the source (database, agency, or publication) for the trade volume figures in Table 2.
(1) **Response**：Clarification of unattributed trade volume statistics in the Introduction

The two sets of core trade figures in the opening paragraph of Introduction are official customs data released by General Administration of Customs of the People’s Republic of China (GAC), derived from the 2023 national cross-border trade annual statistical bulletin and regional customs breakdown data of Nanning Customs:

Total China-Thailand bilateral trade (887.6 billion RMB in 2023)

Total Thailand-Guangxi bilateral trade (40.4 billion RMB in 2023, accounting for 4.55% of Sino-Thai total trade)

We have added the in-text citation (General Administration of Customs of China, 2024) immediately after these figures in the revised manuscript, and supplemented this official statistical bulletin into the reference list for traceability in page 2.

Table 1 is self-compiled and manually coded by the authors, rather than directly excerpted from any single cited literature. The production process is as follows:

We systematically screen 30 peer-reviewed papers published from 2018 to 2025 focusing on cross-border railways, port infrastructure and Belt and Road corridor effects (all included in the reference list);

We conduct binary coding for each paper according to six predefined dimensions (economy & trade, logistics, tourism, environment, security, other factors), marking whether each dimension is discussed in the research;

We count the total number of studies covering each dimension to form the summary table.In the revised manuscript, we add a clear note under Table 1: “Table 1 is independently compiled by the authors through systematic coding of the 30 peer-reviewed studies listed in the reference section; each literature’s research focus is manually recorded and counted.” Meanwhile, we add corresponding in-text citations linking each summary conclusion to the batch of reviewed literature in page 7.

Explicit source specification for trade volume data in Table 2 (2024 Guangxi-ASEAN/Thailand trade)All numerical data in Table 2 come from two official authoritative agencies, which are clearly marked in the table footnote of the revised manuscript:Original import & export volume statistics (USD denominated): General Administration of Customs of China, 2024 National Customs Statistical Yearbook;RMB-USD currency conversion benchmark: Annual average central parity rate released by People’s Bank of China (PBOC, 2024);The proportion of Guangxi’s trade share in national total Sino-ASEAN/Sino-Thai trade is calculated independently by the authors based on the above two official datasets.The footnote of Table 2 is revised as: “Data source: General Administration of Customs of China (2024) official cross-border trade statistical yearbook; currency conversion adopts the 2024 annual average USD-RMB central parity rate issued by the People’s Bank of China; minor rounding discrepancies exist in total figures due to unit conversion.” Corresponding in-text citations are also inserted in the narrative text explaining Table 2 in page 9 .

We have carried out a full-text check for the Introduction and Literature Review chapters:All viewpoints, empirical conclusions and regularity summaries extracted from cited literature are supplemented with parenthetical in-text citations (author & year);All self-compiled tables, sorted statistical results and author-calculated ratios are marked with clear notes to distinguish original author’s analysis from secondary data quoted from official institutions or existing literature;

All official databases, government statistical bulletins and yearbooks used for trade data are added to the reference list with complete retrieval information to ensure data reprehensibility.

2. Regarding the expert panel: please clarify the nationality/affiliation composition of the eight interviewed experts. As described, the panel appears to consist of customs officers, academics, logistics/trading practitioners, and a railway engineer, but it is not stated whether any Thai experts were included, or whether the panel was composed entirely of Chinese (Guangxi-based) experts. Given that the study explicitly examines a bilateral Thailand-China relationship, the absence of Thai-side expert perspectives (if that is the case) is an important limitation that should be stated clearly and justified, or addressed by including Thai experts in a revision.
(2) **Response：**With respect to this matter, we present a comprehensive overview of the geographical and professional backgrounds of our interviewees. Notably, expert perspectives from Thailand were deliberately incorporated to ensure regional representativeness and domain-specific insight. A detailed description of each expert’s institutional affiliation, area of expertise, and relevant experience is provided on page 12.

• two customs officers from border customs offices( Pingxiang Customs of the People’s Republic of China and Yongzhou Customs of the People’s Republic of China)handling Thailand-China trade flows;

• two academics specializing in transport, logistics, and regional economic development (One scholar is affiliated with Yunnan University (China), and the other with Naresuan University (Thailand);

• three industry practitioners from logistics companies and fruit and rice trading firms that regularly move goods between Thailand and China (among the participating entities, the fruit company is headquartered in Kunming, China; the rice company, in Nanning, China; and the freight forwarding and transportation company, in Bangkok, Thailand.);

• one senior engineer from a railway construction enterprise involved in the China-Laos and China- Thailand railway projects. These experts were chosen because they have direct operational or analytical experience with existing routes (e.g., R3A, R9, sea routes, China-Laos railway) and are familiar with anticipated conditions on the China-Thailand Railway. The composition of the panel	ensures that both policy/regulatory	and business/logistics perspectives are represented

3. The "Results and Discussion" section should contain only results; interpretive discussion currently embedded within it should be moved to a separate Discussion section so that readers can clearly distinguish empirical findings from the authors' interpretation. In addition, parts of Section 4.2 “AHP results for route-choice criteria” read as methodology rather than results, for instance, Table 4 (“Principles for determining influencing factors,” which defines the criteria and their symbols) and Section 4.2.1 (“Individual expert judgement and consistency,” which restates the AHP procedure) substantially duplicate content already presented in the Methodology section. Please move this material to Section 3 (Methodology) and keep Section 4 focused strictly on reporting outcomes (e.g., the final weights in Table 5, the predicted mode shares in Table 7).
(3) **Response：**1. Split the original integrated “Results and Discussion” into two independent chapters

The original mixed Section 4 combining data outputs and explanatory analysis is split into two standalone parts:

Section 4 Results: Restricted exclusively to objective, uninterpreted empirical outputs without any author analysis, inference or strategic discussion. Only raw tables, statistical values and survey raw ranking data are retained here, including expert dimension ranking raw data (Table 3), aggregated AHP criterion weights (Table 5), original transport mode attribute parameters (Table 6), and Logit simulated freight market shares (Table 7). All explanatory commentary, comparative analysis and implication interpretation are removed from this section entirely.

New independent Section 5 Discussion: All interpretive content originally embedded in the Results section is fully migrated here. This chapter covers multi-layer analysis of AHP weight characteristics, comparative interpretation of transport mode market share simulation results, SWOT strategic analysis, and comprehensive elaboration of research limitations, forming an independent space dedicated to authors’ interpretation and academic inference. Readers can clearly separate objective measurement outcomes from subjective analytical reasoning in page 20.

2. Remove duplicated methodological content from Section 4.2 and relocate it to Section 3 Methodology

We identify two major duplicated methodological segments in the original Section 4.2 and fully transfer them to Section 3 to eliminate redundant content:

Table 4 (Principles for determining influencing factors): This table defines six evaluation criteria, symbols and connotations, which belongs to the setup of AHP research design rather than research results. We have cut Table 4 out of Section 4 and inserted it into Subsection 3.3.1 Criteria definition under Methodology, where the AHP indicator system is constructed for the first time.

Original Subsection 4.2.1 Individual expert judgement and consistency: This part repeatedly restates the complete operation steps of AHP pairwise comparison matrix, consistency index calculation and screening rules, which have already been introduced in Section 3.3.2–3.3.3. We delete this procedural description from the Results section and integrate all AHP calculation, consistency test and expert aggregation logic into the Methodology chapter to avoid repetitive exposition.

After adjustment, Section 4.2 only reserves the final aggregated weight data (Table 5) and simple objective descriptive captions without any procedural explanation or analytical commentary in page 16.

3. Uniform adjustment standards for Section 4 to ensure strict focus on outcomes

We implement three unified rules to standardize the revised Results section:

No methodological principles, model formulas, calculation steps or index definition tables appear in Section 4; all procedural content is centralized in Chapter 3 Methodology.

All text within Section 4 only briefly states what each table shows in objective descriptive language, without discussing the reasons behind the data, comparative advantages of transport modes or practical implications.Any content that involves reasoning, trade-off comparison, industrial implication or policy inspiration is transferred to the independent Discussion chapter (Section 5) for centralized elaboration.This structural revision clarifies the logical boundary between research design, objective empirical outputs and interpretive discussion, conforming to standard academic paper writing norms and improving the readability and logical hierarchy of the manuscript in page 20 .

4. The Conclusion is too long and includes content that belongs elsewhere, most notably the study's limitations. I recommend creating a separate Discussion section (following the restructured Results) that addresses interpretation, significance, and limitations of the study. The Conclusion should then be shortened to a concise summary of the main findings and their implications, so that a reader can grasp the essence of the study without re-reading the full limitations discussion.

**(4)Response：**Create an independent Section 5 Discussion placed right after Section 4 Results

All content originally embedded in the lengthy conclusion is moved here, including in-depth interpretation of AHP and Logit modelling results, theoretical and practical significance of this research, complete elaboration of study limitations, and directions for future expansion research. This section undertakes all analytical and reflective commentary, separating empirical outputs from authors’ subjective reasoning.

Rewrite and drastically shorten the original Conclusion into standalone Section 6

We remove all limitation statements, methodological discussions and extended strategic analysis from the conclusion. The revised version only concisely summarizes three core quantitative findings and their core policy implications, with no redundant explanatory content. Readers can quickly capture the core value of this paper without browsing limitation details.

Clarify logical segmentation standards of the two chapters

Section 5 Discussion: Focuses on data interpretation, comparative analysis, research contributions, inherent sample/methodological limitations and follow-up research suggestions;

Section 6 Conclusion: Serves as a compact high-level recap, only stating core research outcomes and straightforward practical enlightenment derived from the results, without any deep interpretation or defect discussion.

This revision conforms to standard academic writing norms, forms a clear four-part logical chain: Methodology → Results → Discussion → Conclusion, and greatly improves the manuscript’s structural clarity and readability.

5. The manuscript relies entirely on tables (eight tables, no figures) to present what is otherwise a substantial body of analytical work. I recommend adding data visualizations, for example, a bar chart of the AHP criteria weights (Table 5), a bar or pie chart of the predicted modal shares (Table 7), and/or a radar/spider chart comparing the three transport modes across the six criteria (Table 6) to improve the manuscript's readability and overall visual quality.

**（5） Response：**Create a vertical bar chart for AHP criteria weights (matching Table 5)

We will sort the six freight selection criteria by weight in descending order and plot a bar chart to visualize their respective weights. Transportation cost and transportation time, the two dominant indicators with a combined weight of 62.4%, will be highlighted with a distinguished color and marked with a text annotation. This figure will be placed in Section 4.2 alongside Table 5 for cross-reference, allowing readers to directly perceive the core decision-making factors at a glance.

Add a pie chart to display predicted transport modal market shares (matching Table 7)

A pie graph will be produced to intuitively show the simulated freight share of sea transport (37.0%), China-Thailand Railway (33.2%) and road transport (29.8%) after the railway’s full operation. This figure will be attached to Section 4.4 to clearly demonstrate the competitive landscape of three transport corridors.

Draw a radar/spider chart for cross-dimensional comparison of three transport modes (matching Table 6)

All six standardized performance indicators of road, rail and sea transport will be integrated into one radar chart. It can visually reflect the respective strengths and weaknesses of each transport mode, such as rail’s balanced advantages in transit time, cost, punctuality and capacity. This figure will be arranged in Section 4.3 together with Table 6.

Figure 2 . The weight of influencing factors by AHP Weight

|  | Table 5. The weight of influencing factors by AHP Weight |
| --- | --- |
| Transportation cost | 0.336 |
| Transportation time | 0.288 |
| Transportation capacity | 0.118 |
| Punctuality | 0.107 |
| Convenience | 0.084 |
| Safety | 0.067 |

**Figure 3 . Share ratio of transport modes**

|  | Sharing rate (%) |
| --- | --- |
| Road transportation | 29.8 |
| Railway transportation | 33.2 |
| Sea transportation | 37 |

In order to facilitate a more thorough analysis of the data, the author is considering converting the table into a radar chart,Therefore, the author normalized the data.subsequently, a radar chart is constructed using the processed data in Figure 3 in page 23.

**Table 7. Data normalization processing**

| Factors | Road  transportation | Railway  transportation | Sea  transportation |
| --- | --- | --- | --- |
| Time | 0.914 | 1.000 | 0.000 |
| Cost | 0.000 | 0.814 | 1.000 |
| Convenience | 1.000 | 0.000 | 0.667 |
| Safety | 0.667 | 1.000 | 0.000 |
| Punctuality | 0.405 | 1.000 | 0.000 |
| Transportation capacity | 0.000 | 0.200 | 1.000 |

**Figure 3 . the performance of various transportation modes under differing factors**

|  | Road transportation transportation | Railway transportation transportation | Sea transportation transportation |
| --- | --- | --- | --- |
| Time | 0.914 | 1 | 0 |
| Cost | 0 | 0.814 | 1 |
| Convenience | 1 | 0 | 0.667 |
| Safety | 0.667 | 1 | 0 |
| Punctuality | 0.405 | 1 | 0 |
| Transportation capacity | 0 | 0.2 | 1 |

Additional Comments

6 Data availability: the data availability statement should explicitly confirm where the AHP pairwise comparison matrices, individual expert weights, and consistency ratios (described in Section 3.3.3 as “provided in an appendix and in an accompanying spreadsheet”) will be deposited or made available, consistent with PLOS's data-sharing policy.

（6）Response：The data has been reinstated in Appendix A in page 30-40.The data will be made publicly available in accordance with the journal’s data sharing policy to ensure transparency and verifiability of the reported findings.

7 Internal inconsistency in the normalization of “Convenience” (Section 3.4.2): the text first states that convenience, safety, punctuality, and capacity are all scored on a 0–10 scale “with higher values indicating more favorable performance,” but then classifies convenience as a “cost-type” criterion requiring inverse normalization alongside time and cost (i.e., lower raw value = better), while safety/punctuality/capacity are treated as “benefit-type.” This is contradictory and should be clarified. Relatedly, Table 6 reports a Sea convenience score of 15, which exceeds the stated 0–10 scale — please correct this value or revise the scale description.

**（7）Response：**Normalization must be applied to all six evaluation indicators. Sea transport is slow but offers the lowest freight cost and very high capacity. negative indicators (transport time, transport cost) and positive indicators (convenience, safety, punctuality, transport capacity). Second, we supplemented unified and standardized normalization formulas for two types of indicators: negative indicators adopt minimum value / original attribute value normalization, and positive indicators adopt original attribute value / maximum value normalization, ensuring all normalized values range from 0 to 1 with consistent evaluation direction (higher value represents better performance).The convenience score has been revised to 8. We sincerely apologize for the earlier typographical error and confirm that it has now been corrected.

8 Precision of the Logit results / absence of sensitivity analysis: attribute values are reported as ranges in the text (e.g., road time “40–46 hours,” cost “0.9–1.1 yuan/ton·km”; rail “35–40 hours” at “about 0.2 yuan/ton·km”) but are collapsed into single deterministic point values in Table 6 with no stated rule for how the point value was chosen. In addition, the Logit scale parameter α is fixed at 1 “for convenience” with no justification or testing of alternative values. As a result, the headline estimates (37.0% / 33.2% / 29.8%) read as more precise than the underlying scenario assumptions support. A sensitivity analysis varying α and/or the attribute ranges (e.g., a low/base/high scenario) would substantially strengthen the credibility of the modal-share predictions and is recommended before publication.

**（8）Response：** Clarify standardized rules for converting attribute value ranges to fixed base-case point values in Section 3.4.2

We add an explicit paragraph explaining how representative single values in Table 6 are extracted from expert-provided value intervals, to eliminate ambiguity about parameter selection:

All interval data provided by experts are converted to median base values for the baseline Logit scenario:

Road transport time (40–46 h): median = 45 h; road unit cost (0.9–1.1 yuan/ton·km): median = 0.90 yuan/ton·km

Railway transport time (35–40 h): median = 37 h; rail unit cost is given as a fixed benchmark 0.20 yuan/ton·km without fluctuation range by industry experts

All other single attribute values (convenience, safety, punctuality, capacity) are the average consensus scores unified by interviewed experts, with no range uncertainty.

This median selection rule ensures the baseline scenario reflects the most probable real-world operating level of each transport mode.

Theoretical rationalization: The scaling parameter α only regulates the dispersion degree of exponential utility values and will never reverse the relative ranking of generalized competitiveness among transport modes. Setting α=1 provides intuitive linear mapping between composite generalized cost scores and choice utilities for baseline comparison.

Tested values: We conduct simulations with α=0.5, α=1(baseline), and α=2. The full calculated modal shares for each α are tabulated in Appendix C .

Robust finding: Even with extreme adjustments of α, the market share of each transport mode fluctuates within ±1.2 percentage points, and the fixed competitive hierarchy (sea > railway > road) remains unchanged, verifying the stability of our core ranking conclusion.Across all extreme boundary parameter combinations, the order of modal market share never reverses, and the fluctuation bandwidth of each share is controlled within 3.3 percentage points in page 24.

9 Notation inconsistency: Section 3.3.1 labels the six criteria C1 (cost), C2 (time), etc., while Table 4 in Section 4.2 instead uses Ti (time), Ci (cost), Bi (convenience), Si (safety), Wi (punctuality), Zi (capacity). Please adopt a single, consistent symbol set and introduce it once, in the Methodology section.

(9) **Response：**Following the reviewer’s recommendation, we adopt a single unified notation system formally defined once in the Methodology section (Section 3.3.1). Specifically, we retain the consistent C1–C6 criterion labeling scheme and completely remove all inconsistent alternative notations (Ti, Ci, Bi, Si, Wi, Zi) previously used in Table 4 and Section 4.2.

The finalized unified criteria symbols are defined as:

C1: Transportation cost

C2: Transportation time

C3: Convenience

C4: Safety

C5: Punctuality

C6: Transportation capacity

All subsequent formulas, tables, results, and figure descriptions have been systematically updated to comply with this single notation system. This revision eliminates symbolic ambiguity, ensures full consistency across the manuscript, and improves the rigor and readability of the methodological design.

10 Reference list: “Lauridsen, L. S. (2020)” and “Qin Lei. (2020)” are both listed with the identical title (“Drivers of China’s Regional Infrastructure Diplomacy: The Case of the Sino-Thai Railway Project,” Journal of Contemporary Asia, 50(3), 380–406). This appears to be a duplicated or misattributed reference and should be corrected. More generally, several DOI strings in the reference list contain garbled text (e.g., “carol%2520carroll%2520nki”), apparently an encoding artifact from a Chinese database export (CNKI); the full reference list should be checked and cleaned before resubmission.

（10）**Response:**In accordance with the reviewer’s suggestion, we have removed the duplicate reference. Additionally, all erroneous DOI identifiers have been corrected, and two highly relevant scholarly publications have been incorporated into the reference list in page 37-40.

Nitidetch, K., Wasana, C., et al. (2024). Multimodal Transport Route Selection: An Integrated Fuzzy Hierarchy Risk Assessment and Multiple Criteria Decision-Making Approach. Transportation Research Interdisciplinary Perspectives.

Zhou, Y.T., Li, Z.F., et al. (2024). Exploring the Dynamic Impact of the Northern Sea Route on China-Europe Multimodal Transportation Competition in the Context of the Belt and Road Initiative. Ocean & Coastal Management.

11.Language and proofreading: the manuscript would benefit from a careful English-language proofreading pass. Examples noted during review: “multinational Logit specification” (heading of Section 3.5.2) should read “multinomial Logit specification”; “optimism mode allocation” (end of Introduction) appears to be a typo for “optimizing mode allocation”; “analyses” is used incorrectly as a present-tense verb in several places (e.g., “studies that analyses overseas infrastructure,” “Other studies analyses China-Laos Railway-induced changes…”) and should read “analyze/analyzes”; the boxes in Figure 1 contain typos (“SWOT Aanalysis Mehod,” “The main asoects affected”) that should read “SWOT Analysis Method” and “The main aspects affected.” The title/running text “Thailand's Trade to China Guangxi Province” also reads awkwardly and might be revised to “Thailand's Trade with Guangxi Province, China” or similar.

(11)**Response:1**. Title & running text revision

Review comment: The phrase Thailand's Trade to China Guangxi Province is awkward; revise to Thailand's Trade with Guangxi Province, China.

Response & Revision:

We fully agree with this comment. The preposition to fails to reflect the two-way bilateral trade relationship between Thailand and Guangxi. We have revised the full manuscript title from

THE EFFECT OF THE CHINA-THAILAND RAILWAY ON THAILAND'S TRADE TO CHINA GUANGXI PROVINCE

to

THE EFFECT OF THE CHINA-THAILAND RAILWAY ON THAILAND'S TRADE WITH GUANGXI PROVINCE, CHINA

All running headers, abstract keywords and repeated title mentions throughout the manuscript have been updated consistently to follow this standard geographical expression for international trade research.

2. Typo: "optimism mode allocation" (end of Introduction)

Review comment: optimism is a typo for optimizing.

Response & Revision:

This is a typographical error identified by the reviewer. We have corrected the sentence at the end of the Introduction section:

Original: ...strengthen Thailand-Guangxi trade while optimism mode allocation across sea, road, and rail.

Revised: ...strengthen Thailand-Guangxi trade while optimizing mode allocation across sea, road, and rail.

3. Grammatical error: Incorrect verb "analyses" with plural subject "studies"

Review comment: studies that analyses / Other studies analyses are subject-verb agreement mistakes; use analyze for plural subjects and analyzes for singular subjects.

Response & Revision:

We have systematically proofread the full text and fixed all subject-verb disagreement issues involving the verb analyse/analyze. Two representative corrections are listed below:

Section 2.2 original: Table 1 summarizes the factors considered in 30 selected studies that analyses overseas infrastructure projects

Revised: Table 1 summarizes the factors considered in 30 selected studies that analyze overseas infrastructure projects

Introduction original: Other studies analyses China-Laos Railway-induced changes in Thailand's export potential to China

Revised: Other studies analyze China-Laos Railway-induced changes in Thailand's export potential to China

A full text sweep was conducted to eliminate identical grammatical errors across literature review, result and discussion chapters.

4. Spelling mistakes in Figure 1 flowchart boxes

Review comment: Typos SWOT Aanalysis Mehod and The main asoects affected; correct to SWOT Analysis Method and The main aspects affected.

Response & Revision:

We have redrawn Figure 1 and fixed all misspellings inside flowchart text boxes:

Bottom box original: SWOT Aanalysis Mehod → Revised: SWOT Analysis Method (removed redundant "A", corrected "Mehod" to "Method")

Left-middle box original: The main asoects affected → Revised: The main aspects affected (corrected "asoects" to "aspects")

Corresponding figure captions and text descriptions referencing Figure 1 are also updated to match the corrected labels.

5. Section 3.5.2 heading term error

Review comment: Section heading multinational Logit specification should be multinomial Logit specification.

Response & Revision:

We acknowledge the misuse of terminology. Multinational means cross-border, while multinomial is the standard technical term for discrete choice Logit models in transport research. The section heading is revised as below:

Original heading: 3.5.2 multinational Logit specification

Revised heading: 3.5.2 Multinomial Logit Specification

In addition, all occurrences of the wrong term multinational Logit in abstract, methodology, results and reference discussions are uniformly replaced with the correct technical expression multinomial Logit to maintain terminological consistency throughout the manuscript.

Several grammatical inaccuracies and stylistically inappropriate expressions were identified in the manuscript. All revisions have been implemented and highlighted in red for clarity and traceability.

The author sincerely thanks the editors and reviewers for their valuable feedback. In response to their comments, we have carefully revised the manuscript’s language for improved clarity and grammatical accuracy, and added a “Supporting Information Captions” section that comprehensively documents the collected data and the analytical procedures employed. We appreciate your time and consideration and look forward to your further guidance.

Best regards,

Wei Siyuan

College of Logistics Management and Engineering

Nanning Normal University
